# Characterization of phyto-components with antimicrobial traits in supercritical carbon dioxide and soxhlet *Prosopis juliflora* leaves extract using GC-MS

**DOI:** 10.1038/s41598-023-30390-9

**Published:** 2023-03-11

**Authors:** Nagaraj M. Naik, M. Krishnaveni, M. Mahadevswamy, M. Bheemanna, Udaykumar Nidoni, Vasant Kumar, K. Tejashri

**Affiliations:** 1 Pesticide Residue and Food Quality Analysis Laboratory, University of Agricultural Sciences, Raichur, India; 2 Department of Agricultural Microbiology, University of Agricultural Sciences, Raichur, India; 3 Department of Processing and Food Engineering, University of Agricultural Sciences, Raichur, India

**Keywords:** Microbiology, Plant sciences

## Abstract

This study aimed to screen the bioactive compounds from *Prosopis juliflora* leaf supercritical fluid extract and to assess its antimicrobial properties. Supercritical carbon dioxide and Soxhlet methods were used for extraction. The extract was subjected to Gas Chromatography-Mass Spectrometer (GC-MS) and Fourier Transform Infrared for the characterization of the phyto-components. When compared to soxhlet extraction, more components (35) were eluted by supercritical fluid extraction (SFE), according to GC-MS screening. *Rhizoctonia bataticola, Alternaria alternata,* and *Colletotrichum gloeosporioides* were all successfully inhibited by *P. juliflora* leaf SFE extract, which demonstrated strong antifungal properties with mycelium percent inhibition of 94.07%, 93.15%, and 92.43%, respectively, compared to extract from Soxhlet, which registered 55.31%, 75.63% and 45.13% mycelium inhibition respectively. Also, SFE *P. juliflora* extracts registered higher zone of inhibition 13.90 mm, 14.47 mm and 14.53 mm against all three test food-borne bacterial pathogens viz *Escherichia coli, Salmonella enterica* and* Staphylococcus aureus *respectively*.* Results obtained from GC-MS screening revealed that SFE is more efficient than soxhlet extraction in recovering the phyto-components. *P. juliflora* may provide antimicrobial agents, a novel natural inhibitory metabolite.

## Introduction

Chemical diversity has a variety of benefits for the crucial and difficult subject of discovering innovative medications. New drugs may be created from biomolecules or phytochemicals present in natural products like plant extracts. There are a variety of chronic and infectious diseases that can be cured using the numerous active components present in plants used in traditional medicine^1^. Thousands of phytochemicals are having antimicrobial, antioxidant, wound headlining, anticarcinogenic and antidiarrheal traits. Many studies reported plants as safe and broadly effective alternatives with fewer adverse effects^1,2^.

The shrub *P. juliflora* is known to contain different chemical compounds such as alkaloids, flavonoids, terpenoids, saponins, and phenolic compounds distributed in different parts of the plant body which makes the shrub of medicinal importance. *P. juliflora* (Sw.) DC, (Velvet Mesquite) contains 44 species, of which 40 are native to America, three to Asia, and one to Africa and belong to the family Fabaceae, subfamily Mimosoideae^3^. Medically active substances have been extracted from different parts of *P. juliflora* such as leaves, roots, stems, branches, bark as well as pollen. Crude extract from *P. juliflora* known to contain a varied class of secondary metabolites which has unique and combined therapeutic and antifungal traits^4–6^. The usage of this plentiful resource imparts a good option for yielding bioactive natural products that may serve as an important raw material for the pharmaceutical and chemical industries^7^.

The selection of the use of solvent and the extraction procedure in obtaining the antimicrobial metabolites from various plant parts play a major role^8^. There are various conventional extraction methods to obtain an extract from the plant. Among the techniques, soxhlet has been mostly used for a long time^8^. However, conventional extraction methods have the considerable drawback of solvent residue leftover in the extracts, time consumption and poor recovery. The new technique of Supercritical Fluid Extraction (SFE) has secured prime attention over the traditional techniques in the recovery of edible and essential oils in the field of natural products. Supercritical fluids extraction (SFE), or the extraction of components using solvents at high pressure, has attracted a lot of attention in recent years and has been used for a variety of applications, particularly in the food, pharmaceutical, and cosmetic industries^9^. Supercritical carbon dioxide (SC-CO2) is especially popular due to its inertness, non-toxicity, non-flammability, and low cost. SC-CO2 processes can be carried out at low temperature that results in preserving the original oil compositions and properties^10^. Supercritical fluid extraction (SFE), especially by supercritical carbon dioxide (SC-CO2) technique has been known as an alternative suitable tool for the extraction of essential oil and seed oil from various plants^11^. Pressurized fluids are used as solvents in the extremely selective SFE technique. When a fluid is driven to temperatures and pressures over its critical point, the liquid and gas phases become indistinguishable from one another, creating a supercritical fluid. At optimal conditions, SFE does not have any of the adverse effects of traditional organic solvents. Optimization of temperature and pressure has a major impact on fluid density, improved transport properties, higher extraction yield, and shorter extraction time in SFE. The most commonly used supercritical fluid (SF) is SC-CO_2_ because it has a moderate critical temperature (31.3 °C) and pressure (72.9 atm). Giving high-quality, solvent-free extracts at low temperatures, SC-CO2 extraction allows active compounds to be protected from thermal degradation, while providing more selective and efficient extraction by controlling the process temperature and pressure, so as to adjust CO2 density hence regulating the solvating power. The ability to control the extraction parameters is the major benefit of the supercritical fluid extraction process^12–15^.

After the extraction, it is very important to determine the components present in the extract. The application of chromatographic techniques is most suitable for the determination of various phytoconstituents (qualitatively and quantitatively) present in the extract. Gas chromatography-mass spectrometry (GC-MS) is a combined analytical technique used to determine and identify compounds present in any matrix sample. GC-MS with triple quadrupole is suitable equipment for an important role in the analysis of bioactive components and chemotaxonomic studies of medicinal plants containing biologically active components^16^.

There hasn't been any research published to date on the comparison of extraction techniques like SFE and Soxhlet through GC-MS analysis of the bioactive components of *P. juliflora* leaf extract. The Pesticide Residue and Food Quality Analysis Laboratory at the University of Agricultural Sciences, Raichur, Karnataka, India conducted research intending to characterize Phyto-components with antifungal traits present in he supercritical fluid and soxhlet *P. juliflora* leaves extract using GC-MS while keeping these facts in mind (Supplementry Table 1).

## Results and discussion

### Extraction yield and efficiency of *P. juliflora* leaf extract

The extraction yield and extraction efficiency of 14.10 g/100 g and 93.37% were recorded at SC-CO_2_ extraction whereas Soxhlet extraction registered 9.25 g/100 g and 61.25% respectively (Table 1). Based on the previous experiment result obtained, a pressure of 200 bar, and a temperature of 50 °C were considered as the optimum and best SC-CO2 extraction condition for obtaining the highest extraction efficiency from *P. juliflora* leaf powder. It is evident from the result that higher extraction yield and efficiency were found in SC-CO2 extraction compared to Soxhlet extraction. This might be since the increase in pressure increases the density of the CO_2_ thereby increasing the solvent strength and solubility of the oil in CO_2_. Naturally, a raise in pressure at a given temperature increases SC-CO2 density, hence enhancing its solubility. The diffusion coefficient is also lowered as a result of this action. This may be due to the comforting solute-solvent ejection brought on by the extremely compressed CO2 ^17–21^.

**Table 1:** Extraction yield and extraction efficiency of *P. juliflora* leaf extract.

Extraction method	Extraction yield (g/100 g)	Extraction efficiency (%)
SC-CO2	14.10	93.37
Soxhlet	9.25	61.25
SEm ±	0.13	0.11
CD @ 1%	0.55	0.47

### Gas chromatography-mass spectrometry triple quadrupole (GCMS-MS) analysis

**Figure 1:**
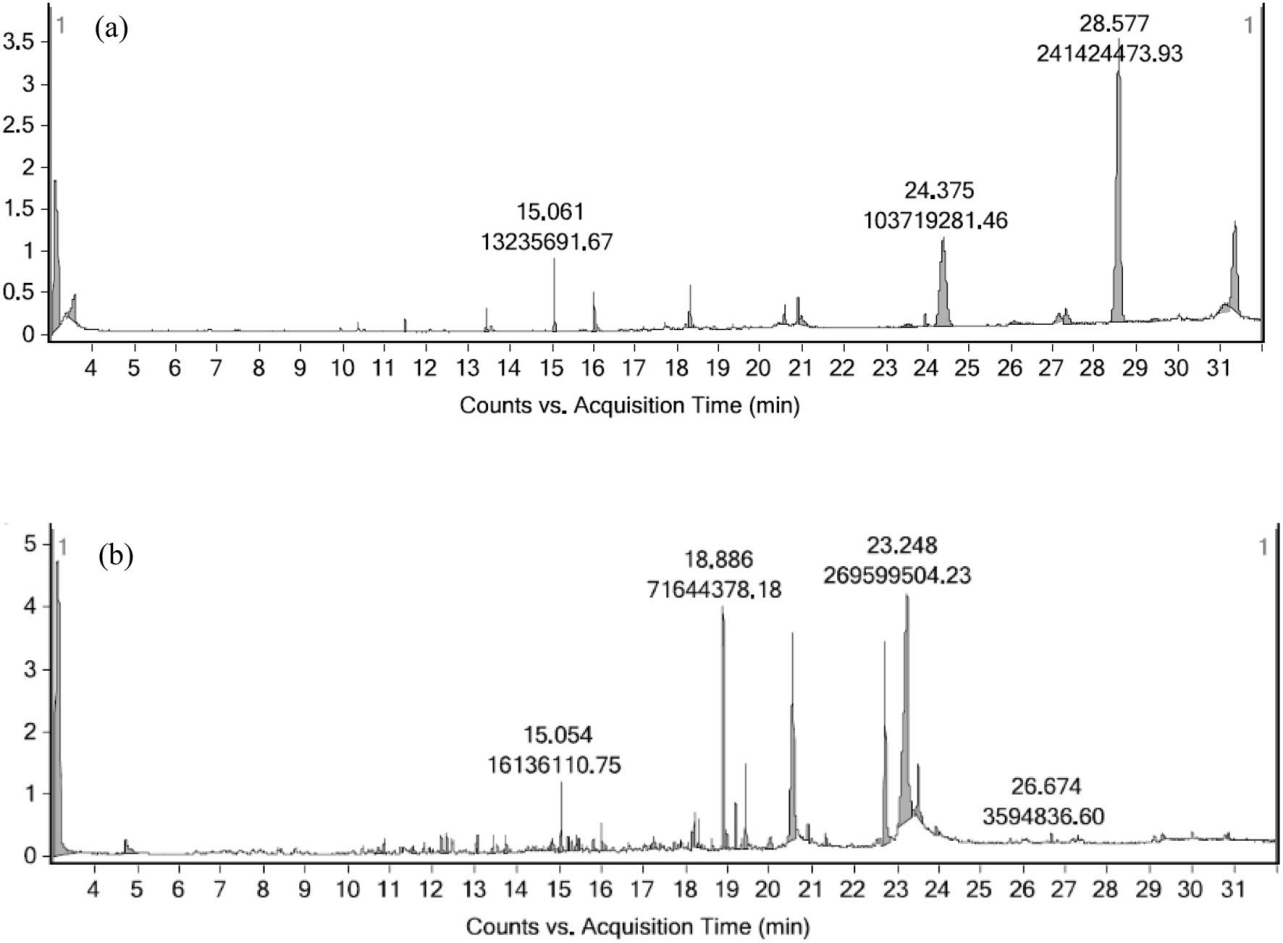
GCMS-MS chromatogram (**a**) Soxhlet extract (**b**) SFE extract.

As expected, a wide variety of bioactive compounds could be found in Soxhlet and SFE extract. Twenty compounds were detected from the GCMS-MS analysis of soxhlet extract of *P. juliflora* leaves whereas thirty-five compounds were identified from the GCMS-MS analysis of a supercritical fluid extract of *P. juliflora* leaves. The chromatogram is depicted in Fig. 1, while the name of bioactive components with their retention time (RT), molecular formula, height, and area are presented in Tables 2 and 3. The major compounds identified in the *P. juliflora* soxhlet extract are Phenol, 3,5-bis(dimethyl ethyl), Benzene dicarboxylic acid, and Squalene. The major components detected in SFE extract of *P. juliflora* leaves are Pentanoic acid 5hydroxy 2,4, dibutyl phenyl ester, Phytol, Tetramethyl heptadecane, Neophytadiene, hexadecanal. Many other compounds were traced as low levels. All these major plant metabolites have a role as anti-inflammatory agents, anti-oxidants, and antimicrobial agents. More compounds are eluted in SFE extract compared to Soxhlet extract may be due to the special features of SFE. The unique characteristics of SFE, such as excellent extraction efficiency and selectivity, are caused by its liquid-like solubility and gas-like mass transport characteristics. Extraction of analytes present in low concentrations, cleaner extracts, and preservation of bioactive constituents could be achieved through SFE^22^. Even higher extraction yield can be achieved by providing close contact between the sample and extractant.

**Table 2:** Bioactive components with retention time (RT), molecular formula, height, and area of Soxhlet *P. juliflora* extract.

Sl. no.	Compound label	RT	Formula	Height	Area
1	Ethyl Acetate	3.112	C_4_H_8_O_2_	18029329	112121755
2	Butanoic acid	3.591	C_4_H_8_O_2_	3404926	20695565
3	Acetic acid, 2-phenylethyl ester	11.48	C_10_H_12_O_2_	1546234	2540370
4	1-Tetradecene	13.43	C_14_H_28_	2887070	4421657
5	Phenol, 3,5-bis(1,1-dimethylethyl)-	15.06	C_14_H_22_O	8732153	13235692
6	1-Hexadecanol	16.01	C_16_H_34_O	4684995	6681963
7	2-Piperidinone, N-[4-bromo-n-butyl]-	18.31	C_9_H_16_BrNO	5038996	10916233
8	Phthalic acid, butyl tridec-2-yn-1-yl ester	20.57	C_25_H_36_O_4_	2281893	4653279
9	1-Decanol, 2-hexyl-	20.90	C_16_H_34_O	3221931	7927989
10	2-Methyltetracosane	20.98	C_25_H_52_	1149186	6149745
11	7-Hexadecenal, (Z)-	23.51	C_16_H_30_O	381911	3442115
12	5-Eicosene, (E)-	23.94	C_20_H_40_	1579729	3884158
13	1,4-Benzenedicarboxylic acid, bis(2-ethylhexyl) ester	24.37	C_24_H_38_O_4_	10537558	103719281
14	7-Hexadecenal, (Z)-	26.11	C_16_H_30_O	435929	3787460
15	1-Decanol, 2-hexyl-	27.14	C_16_H_34_O	1059557	7287544
16	Carbonic acid, eicosyl vinyl ester	27.29	C_23_H_44_O_3_	1856158	12056569
17	Squalene	28.57	C_30_H_50_	34034322	241424474
18	7-Hexadecenal, (Z)-	29.48	C_16_H_30_O	348577	3521799
19	Benzeneethanol, .beta.-methoxy-.beta.-(trifluoromethyl)-, (S)-	31.10	C_10_H_11_F_3_O	1127897	11254787
20	Heneicosane	31.35	C_21_H_44_	10675295	77709695

**Table 3:** Bioactive components with retention time (RT), molecular formula, height, and area of SFE *P. juliflora* extract.

Sl. no.	Compound label	RT	Formula	Height	Area
1	Ethyl Acetate	3.11	C_4_H_8_O_2_	47481915	335863804
2	p-Xylene	4.734	C_8_H_10_	2047946	13150162
3	2,4,4-Trimethyl-1-pentanol, 2- methylpropionate	10.72	C_12_H_24_O_2_	1022902	4743155
4	Benzaldehyde, 2,4-dimethyl-	10.86	C_9_H_10_O	2364457	4564856
5	2-Bromotetradecane	11.54	C_14_H_29_Br	1190642	3445335
6	2-Isopropyl-5-methyl-1-heptanol	12.20	C_11_H_24_O	2967242	6067812
7	2-Isopropyl-5-methyl-1-heptanol	12.33	C_11_H_24_O	3149993	6015443
8	Naphthalene, 1,2,3,4-tetrahydro-1,1,6- trimethyl-	13.07	C_13_H_18_	3034830	5203641
9	1-Tetradecene	13.42	C_14_H_28_	2895992	4230978
10	Naphthalene, 1,5-dimethyl-	13.73	C_12_H_12_	2967620	5952515
11	Heptadecane, 2,6,10,15-tetramethyl-	14.83	C_21_H_44_	1711282	4427511
12	Pentanoic acid, 5-hydroxy-, 2,4-di-tbutylphenyl esters	15.05	C_19_H_30_O_3_	11211181	16136,111
13	1,3-Butanedione, 1-phenyl-	15.22	C_10_H_10_O_2_	2199453	5042320
14	Nonadecane	15.39	C_19_H_40_	2420507	4941251
15	5-Nitro-4,6-pyrimidinediol	15.82	C_4_H_3_N_3_O_4_	2143562	5595180
16	1-Hexadecanol	16.00	C_16_H_34_O	4547223	6717343
17	1-Octadecanesulphonyl chloride	17.23	C_18_H_37_ClO_2_S	1762504	5391876
18	1,4-Naphthalenedione, 2,3,6-trimethyl-	18.16	C_13_H_12_O_2_	2551,195	4274909
19	6-Hydroxy-4,4,7a-trimethyl-5,6,7,7atetrahydrobenzofuran- 2(4H)-one	18.21	C_11_H_16_O_3_	5829,047	10165591
20	1-Nonadecene	18.31	C_19_H_38_	4635481	7611077
21	Neophytadiene	18.88	C_20_H_38_	39026264	71644378
22	Phytol	18.96	C_20_H_40_O	3027518	5477980
23	3,7,11,15-Tetramethyl-2-hexadecen-1- ol	19.18	C_20_H_40_O	7543136	13776914
24	Neophytadiene	19.42	C_20_H_38_	13242210	25270940
25	L-Lysine, N(6)-[(1,1-dimethylethoxy)carbonyl]-N(2)-[(9Hfluoren- 9-ylmethoxy)carbonyl]-	20.00	C_26_H_32_N_2_O_6_	1982886	9153357
26	n-Hexadecanoic acid	20.54	C_16_H_32_O_2_	33264532	146691362
27	11-Methyldodecanol	20.90	C_13_H_28_O	2574190	5428167
28	Oxirane, hexadecyl-	21.31	C_18_H_36_O	2036065	3927815
29	Phytol	22.72	C_20_H_40_O	32397714	90470552
30	9,12,15-Octadecatrienoic acid, (Z,Z,Z)-	23.24	C_18_H_30_O_2_	36602649	269599504
31	9,12-Octadecadienoic acid (Z,Z)-	23.50	C_18_H_32_O_2_	8490307	26042217
32	7-Hexadecenal, (Z)-	23.93	C_16_H_30_O	1287334	3935590
33	4,8,12,16-Tetramethylheptadecan-4- olide	26.67	C_21_H_40_O_2_	1417112	3594837
34	7-Hexadecenal, (Z)-	29.30	C_16_H_30_O	952950	4230944
35	Tetradecane, 2,6,10-trimethyl-	30.85	C_17_H_36_	1067075	3518551

### FTIR spectroscopy analysis of *P. juliflora* leaves extract

**Figure 2:**
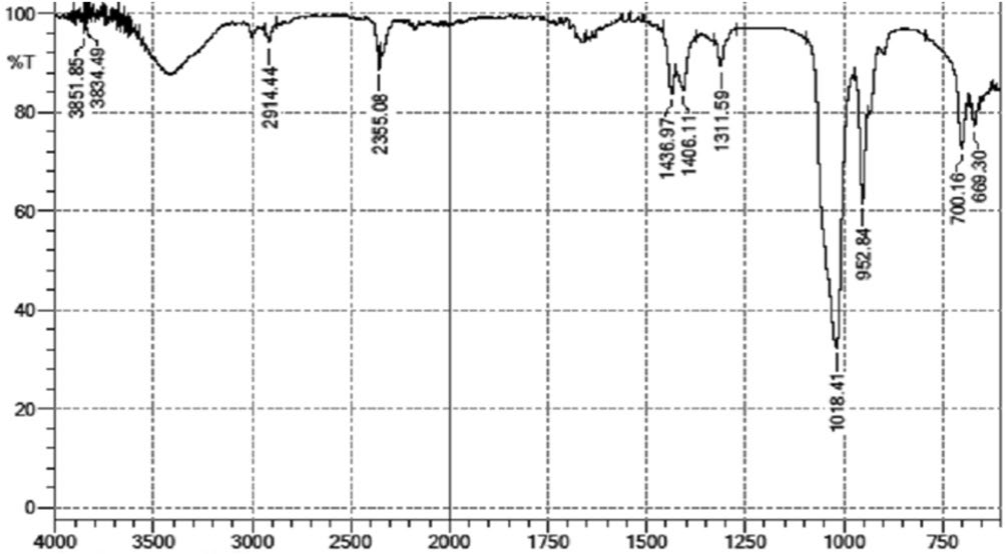
FTIR spectra of *P. juliflora* leaves SFE extract.

Phytochemical screening is an important step that leads to the isolation of new and novel compounds. The results of FTIR analysis of the *P. juliflora* leaf SFE extract revealed the presence of different functional groups in the extract (Fig. 2). Major peaks in the FTIR analysis showed the presence of alcohol, phenols, alkanes, aromatic, ether, carboxylic acid, aliphatic amines, primary and secondary amine. Similar preliminary phytochemical screening of the *P. juliflora* extract through FTIR also revealed that the plant contains Alkaloids, Flavonoids, Saponins, Tannins, Anthraquinone Glycoside, and Coumarins^23,24^.

### Antimicrobial activity of *P. juliflora* leaf extract

The antimicrobial activity of Soxhlet and SC-CO_2_ *P. juliflora* leaf extract against the fungal plant pathogens viz., *Rhizoctonia bataticola, Alternaria alternata* and *Colletotrichum gloeosporioides* and food borne bacterial pathogens viz *Escherichia coli, Salmonella enterica and Staphylococcus aureus* is presented in Tables 4 and 5. SFE *P. juliflora* extract registered higher zone of inhibition 94.07%, 93.15% and 92.42% against all three test pathogens *Rhizoctonia bataticola, Alternaria alternata and Colletotrichum gloeosporioides respectively* whereas Soxhlet *P. juliflora* extract recorded 79.58%, 85.07% and 66.57% zone of inhibition respectively. SFE *P. juliflora* extract registered higher zone of inhibition 13.90 mm, 14.47 mm and 14.53 mm against all three test food-borne bacterial pathogens viz *Escherichia coli, Salmonella enterica* and *Staphylococcus aureus respectively* whereas Soxhlet *P. juliflora* extract recorded 5.10 mm, 5.20 mm and 6.10 mm zone of inhibition respectively. This result demonstrated that SFE extract has more antimicrobial potential compared to Soxhlet extract. Even though the extract was obtained from the same *P. juliflora* leaves, the method applied for extraction play a major role in the recovery of biomolecules from the raw material.

**Table 4:** Antifungal activity of supercritical fluid and soxhlet *P. juliflora *leaf extract against fungal test organisms.

Treatments	*Rhizoctonia bataticola*		*Alternaria alternata*		*Colletotrichum* *gloeosporioides*	
	Mycelia growth (mm)	Inhibition (%)	Mycelia growth (mm)	Inhibition (%)	Mycelia growth (mm)	Inhibition (%)
SFE	5.30^a^	94.07^a^	6.10^a^	93.15^a^	6.37^a^	92.43^a^
Soxhlet	18.10^b^	79.58^b^	13.30^b^	85.07^b^	28.13^b^	66.5^b^
Negative control	90.00	0.00	90.00	0.00	88.00	0.00
Positive control	5.00^a^	94.60^a^	4.90^a^	94.57^a^	5.10^a^	93.77^a^
SEm ±	0.16	0.20	0.23	0.20	0.29	0.27
CD @1%	0.76	0.97	1.11	0.97	1.39	1.30

*SFE* Supercritical fluid extraction.

Significant values are in [superscript a, b].

**Table 5:** Antibacterial activity of supercritical fluid and soxhlet *P. juliflora *leaf extract against bacterial test organisms.

**Treatments**	**Zone of inhibition (mm)**		
	***Escherichia coli***	***Salmonella enterica***	***Staphylococcus aureus***
SFE	13.90_b_	14.47_a_	14.53_a_
Soxhlet	5.10_h_	5.20_g_	6.10_f_
Negative control	**–**	**–**	**–**
Positive control	15.20_a_	15.60_a_	15.70_a_
SEm ±	0.22	0.23	0.40
CD @ 1%	1.03	1.07	1.91

*SFE* Supercritical fluid extraction.

Significant values are in [subscript a, b, c, d, e, f].

According to Saleh et al.^5^ the water-soluble leaf ethanolic extract of *P. julifora* displayed significant antibacterial activity against *Staphylococcus* sp. and *E. coli*. The results of the present investigation were in agreement with Raghavendra et al*.*^25^ that the activity of the aqueous extract of *P. juliflora* against *Alternaria alternata showed* 71.59% inhibition of mycelial growth. Deressaa and associates^26^ used methanol, acetone and aqueous extract of *P. juliflora* leaves against *Colletotrichum gloeosporioides* the results showed radial growth inhibition of 100 percent, 100 percent, 79.60 percent, respectively. Bazie et al*.*^27^ reported the activity of methanolic extract of *P. juliflora* against *Colletotrichum musae*, which showed a 30.70 mm zone of inhibition.

*P. juliflora* demonstrated significant antimicrobial activity and may be used to identify bioactive natural products that can serve as leads for developing new pharmaceuticals that address previously unmet needs^28^. The results indicate that leaves extracted from *P. juliflora* are a promising source of antimicrobial agents and may have therapeutic potential. A deeper study of this plant with its pure compounds may lead to the development of natural alternative antimicrobial compounds against plant and food borne pathogens. A** s**imilar study was carried out by Rizwana et al.^29^ who isolated two pentanoic acid compounds from *Bluejack Oak* and tested them for their antimicrobial potential and showed promising antifungal activity against *Aspergillus niger* and *Aspergillus flavus*.

## Conclusion

Despite the fact that the extract was produced using the same *P. juliflora* leaves, the method of extraction's efficacy is extremely important. The GC-MS results revealed that the supercritical fluid extraction method is superior to the soxhlet extraction method for removing the bioactive components from *P. juliflora* leaves. Fungal pathogens such as *Rhizoctonia bataticola, Alternaria alternata, and Colletotrichum gloeosporioides* and bacterial food borne pathogens such as *Escherichia coli, Salmonella enterica* and *Staphylococcus aureus* are effectively suppressed by *P. juliflora* SFE extract because it includes antimicrobial substances including Phytol, Tetramethyl heptadecane, Neophytadiene, and Pentanoic acid 5hydroxy 2,4 dibutyl phenyl ester. *P. juliflora* could be a potential source for antimicrobial agents, a novel inhibitory metabolite.

## Materials and methods

### Raw materials

Fresh leaves of *P. juliflora* were collected around the campus of the University of Agricultural Sciences (UAS), Raichur, Karnataka State. Leaves were cut and dried in dehumidified air dryer (make: Bry Air Asia; model: FSD-600) at 45 °C and 15% relative humidity. The dried leaves were ground in a laboratory hammer mill to obtain a fine powder.

### Chemicals

Chemicals used for the analysis included n-hexane (CAS 110-54-3), ethanol (CAS 64-17-5), Phenyl methyl siloxane (CAS CAS 68037-54-7) and Potassium bromide (CAS 7758-02-3). The solvents, chemicals, and reagents (analytical grade) used throughout the experiment were procured from M/s. Sigma Aldrich Chemicals, Germany and Merck, Germany.

### Microbial culture

Authentic pure cultures of bacterial food borne pathogens were procured from American Type Culture Collection (ATCC), USA and Microbial Type Culture Collection (MTCC), Chandigarh, India, namely *Escherichia coli* (ATCC 0680P), *Salmonella enterica* (MTCC 98) *and Staphylococcus aureus* (MTCC 87). Pure fungal cultures of *Rhizoctonia bataticola* (ATCC 26020), *Alternaria alternata* (ATCC 66981)*,* and *Colletotrichum gloeosporioides* (ATCC 20358) were collected from the Department of Plant Pathology, UAS, Raichur. Procured cultures were maintained in the appropriate media for further use.

### Extraction of *P. juliflora* leaf extract

Supercritical fluid and Soxhlet extraction methods were employed for obtaining an extract from *P. juliflora* leaves.

### Supercritical fluid extraction

**Figure 3:**
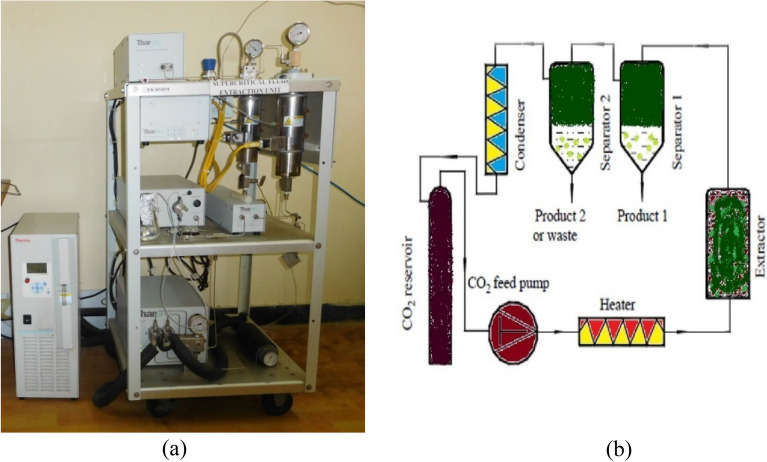
SFE instrument (**a**) and schematic diagram of SC-CO_2_ (**b**).

The supercritical carbon dioxide (SC-CO_2_) extraction system (Thar; SFE 500 system) was used for the extraction of *P. juliflora* leaf powder. Deionized water (5 °C) was used for cooling different zones in the SC-CO_2_ extraction system. Fifty grams of *P. juliflora* leaf powder were placed into the extractor vessel. The flow rates of supercritical CO_2_ and co-solvent (ethanol) were maintained at 20 and 2 g/min, respectively ^30^. The static extraction process was performed for 30 min. After attaining desired pressure (200 bar) and temperature (45^0^C) dynamic extraction time (90 min) was started by opening the exit valve of the SC-CO_2_ extraction system. The static extraction time allowed the sample to soak in the CO_2_ and co-solvent to equilibrate the mixture at desired pressure and temperature. During the dynamic extraction time, CO_2_ carrying the crude extract flowed out of the extraction vessel and then into a collection vessel, where the CO_2_ was separated through the vent connected to the fume hood ^31^. The SFE instrument and scheme diagram of SC-CO_2_ is depicted in Fig. 3.

### Soxhlet extraction

*P. juliflora* leaves extraction was carried out by the soxhlet extraction method using SOCS- PLUS apparatus (Make: Gerhardtz, model: SOX-416) with hexane as solvent. Accurately, 50 g of the *P. juliflora* leaf powder was taken into the thimble and placed in the sample compartment of the extractor. The sample compartment was attached to a 500 ml round bottom flask containing 300 ml hexane. The SOCS- PLUS apparatus was run at 85 °C for 90 min. Hexane in the extract was distilled out by using a rotary flash vacuum evaporator (Superfit, Rotavap; PBU-6D)^32,33^.

*Extraction Yield* The extraction yield was computed by using the following equation

1 $${\text{Extraction yield }}\left( {{\text{g}}/{1}00{\text{g}}} \right) = \frac{{\text{M feed}}}{{\text{M extract}}} \times { 1}00$$

where, M extract = Mass crude extract (g) M feed = Feed mass (g).

*Extraction Efficiency* The extraction efficiency was calculated as per the equation described. It is the ratio of the quantity of extract obtained during the process to the actual amount of extract present in 100 g of P*. juliflora* leaves

2 $${\text{Extraction efficiency }}\left( \% \right) = \frac{{{\text{Actual extarct present}},{\text{ g}}/100{\text{ g of sample}}}}{{{\text{Extarct extracted}},{\text{ g}}/100{\text{ g of sample}}}} \times { 1}00$$

### Preparation of extracts for GC-MS analysis

The extract obtained from both extraction methods were filtered through a 45 μm filter. The resulting solution was concentrated in vacuum to dryness to give a solvent free extract. The extract was stored in a refrigerator at 4 °C for further use.

### GC-MS triple quadrupole analysis

GC-MS analysis was carried out in a combined 7890B gas chromatograph system Agilent make and mass spectrometer triple quadrupole fitted with an HP-5 MS fused silica column (5% phenyl methyl siloxane 30.0 m × 250 µm, film thickness 0.25 µm, interfaced with 7000D Agilent mass detector with (TQD) triple detector. Helium gas was used as carrier gas and was adjusted to column velocity flow of 1.0 ml/min.

Other GC oven conditions are 60 °C at initial temperature at 7 °C/min reaching to 270 °C with spitless, injection volume 1 µl and Mass condition ion source temperature, 280 °C with an injection temperature of 250 °C. The data were integrated using software and compiled with the compounds with the provided NIST Library software to identify unknown compounds and structures.

### FTIR analysis of *P. juliflora* leaf powder extract

Supercritical fluid *P. juliflora* leaves extract was used for FTIR (Fourier Transform Infrared Spectroscopy) analysis with the Attenuated Total Reflectance (ATR) sampling method that introduces light on the sample to acquire structural and compositional information. About mg of the finely ground sample is then placed onto the face of a KBr plate. FTIR analysis was performed using the Shimadzu FTIR spectrometer 8000 series, between 4000 and 750 cm^-1^ which was used to detect the characteristic peaks and their functional groups. The peak values and the functional groups of *P. juliflora* leaf SFE extract were recorded.

### Antifungal activity of *P. juliflora* leaf extract

The antifungal activity of Soxhlet and SFE *P. juliflora* leaf extract against the plant pathogens viz., *Rhizoctonia bataticola, Alternaria alternata,* and *Colletotrichum gloeosporioides* was carried out following poisoned food technique^34^. The potato dextrose agar (PDA) media was prepared and sterilized. Plant extract obtained from both extraction methods was exposed to a rotary flash vacuum evaporator for the complete removal of organic solvents used for the extraction. The extracts were resuspended in distilled water and sterilized by membrane filtration with pore 45 m (Whatman brand) and stored at 4 °C until use. A volume of 0.5 ml of plant extract (10 mg/ml) was aseptically poured into a Petri plate followed by the addition of 9.5 ml of melted PDA and was gently mixed. The inoculum disc of test fungus was aseptically inoculated upside down at the center of the Petri plate and incubated at 25 °C for 7 days.

The media plate with 0.5 ml of sterile distilled water in place of extract (without extract) was set as a negative control and the media plate supplemented with 0.1% hexaconazole was considered a positive control. The inhibition percentage of the fungal mycelia was measured on the 7^th^ day of incubation.

3 $${\text{Mycelial}}\;{\text{inhibition}}\;(\% ) = \frac{{{\text{DC}} - {\text{DT}}}}{{{\text{DC}}}} \times 100$$

where,

DC—Average diameter of the colony in the control plate.

DT—Average diameter of the colony in the treatment plate.

### Antibacterial activity of *P. juliflora* leaf extract

Disc agar diffusion technique was employed for antibacterial bioassay. Petri plates were washed, rinsed with sterile distilled water, dried, wrapped in tin foil and kept in an autoclave at 100 °C for 15 min to sterilize. For testing antimicrobial activity against bacteria 20 ml of growth medium and 4 ml of bacterial inoculum were mixed and poured into separate sterilized Petri plates. Each mixture was thoroughly shaken to ensure uniform distribution of inoculum. The experiment was carried out in three replicates. Sterile paper discs measuring 6 mm in diameter, which absorbs about 0.1 ml of the extract (10 mg/ml) solution were employed for test in test samples. All test petri plates were kept at 5 °C for 40–50 min to allow the diffusion of the substances and then incubated at 35–37 °C for 18 h. The inhibition zones formed by the *P. juliflora* leaf extract were measured including the diameter of the paper disc.

### For experimental research and field studies on plants

All procedures were conducted in accordance to the relevant institutional, national, and international guidelines and legislation.

## Supplementary Information

Supplementary Information.

## Data Availability

All data generated or analysed during this study are included in this published article.
