# Supplementary material for: Characterization of phyto-components with antimicrobial traits in supercritical carbon dioxide and soxhlet Prosopis juliflora leaves extract using GC-MS
Source: Sci Rep. 2023 Mar 11;13:4064. doi: 10.1038/s41598-023-30390-9 (PMC10008579; doi:10.1038/s41598-023-30390-9)
Supplement: Supplementary file 1 — Supplementary Information. [file 41598_2023_30390_MOESM1_ESM.docx]

Supplementary Material

**Table:1 Effect of SC-CO_2_ temperature and pressure on extraction yield and extraction efficiency of *Prosopis juliflora* leaf extract**

| **Treatments** | **Extraction yield (g/100g)** | **Extraction efficiency (%)** |
| --- | --- | --- |
| T1 (Temperature 40 ^0^C and Pressure 100 bar) | 8.40^f^ | 55.62 ^f^ |
| T2 (Temperature 40 ^0^C and Pressure 150 bar) | 9.15**^e^** | 60.59 ^e^ |
| T3 (Temperature 40 ^0^C and Pressure 200 bar) | 12.00^c^ | 79.46 ^c^ |
| T4 (Temperature 50 ^0^C and Pressure 100 bar) | 9.80**^d^** | 64.89 ^d^ |
| T5 (Temperature 50 ^0^C and Pressure 150 bar) | 12.60 ^b^ | 83.44 ^b^ |
| T6 (Temperature 50 ^0^C and Pressure 200 bar) | 14.10^a^ | 93.37 ^a^ |
| T7 (Temperature 60 ^0^C and Pressure 100 bar) | 8.60^f^ | 57.17^f^ |
| T8 (Temperature 60 ^0^C and Pressure 150 bar) | 9.74^d^ | 64.67 ^d^ |
| T9 (Temperature 60 ^0^C and Pressure 200 bar) | 12.70^b^ | 84.10^b^ |
| T10 (Soxhlet extraction) | 9.25^e^ | 61.25 ^e^ |
| **Mean** | **10.64** | **70.49** |
| **SEm±** | **0.070** | **0.440** |
| **CD (1%)** | **0.210** | **1.318** |

T1 to T9: Super critical fluid extraction (SFE) where as T10: Soxhlet extraction

Note: Mean values followed by same superscript letters are not significantly different
